# Assessment of the role of *Haemophilus ducreyi* coinfection on outcomes of Yaws treatment in the southwestern part of Ghana

**DOI:** 10.1371/journal.pntd.0014182

**Published:** 2026-03-30

**Authors:** Abigail Agbanyo, Michael Ntiamoah Oppong, Dzifa Kofi Ahiatrogah, Ruth Dede Tuwor, Clement Tettey, Joseph Azabire, Owusu Boakye Yiadom, Dennis Odai Laryea, Alex Owusu-Ofori, Richard Odame Phillips, Yaw Ampem Amoako

**Affiliations:** 1 Kumasi Centre for Collaborative Research in Tropical Medicine, Kwame Nkrumah University of Science and Technology, Kumasi, Ghana; 2 School of Medical Sciences, Kwame Nkrumah University of Science and Technology, Kumasi, Ghana; 3 Ghana Health Service, Sekondi, Ghana; 4 Wassa Amenfi East District Health Directorate, Ghana Health Service, Wassa Akropong, Ghana; 5 Aowin District Health Directorate, Ghana Health Service, Enchi, Ghana; 6 Disease Surveillance Department, Ghana Health Service, Accra, Ghana; 7 Komfo Anokye Teaching Hospital, Kumasi, Ghana; Fundacao Oswaldo Cruz, BRAZIL

## Abstract

**Background:**

Yaws, caused by *Treponema pallidum* subsp. *pertenue* (TP) is targeted for eradication by 2030 under the World Health Organization’s (WHO) initiative, which relies on mass drug administration (MDA) of azithromycin. Evidence of persistent lesions after treatment has been reported. While the occurrences of *Haemophilus ducreyi* (HD) in yaws-like lesions has been widely documented, there is limited evidence regarding its influence on treatment outcomes assessed at the WHO-recommended 4-week follow-up. In this study, we sought to detect the occurrence of *H. ducreyi* in yaws-confirmed cases and assess its influence on treatment outcomes.

**Methodology:**

We conducted a prospective cohort study of school children from Wassa Amenfi East and Aowin Districts in Ghana. A total of 46 Dual Path Platform (DPP) positive yaws cases were subjected to PCR analysis to determine the aetiological agents, including *T. pallidum* and/or *H. ducreyi.* Treatment was with a single dose of oral azithromycin and outcomes were evaluated 4 weeks post-treatment, assessing clinical resolution and time to healing, with results stratified by the identified aetiological agents.

**Findings:**

Of the 46 participants tested, 18/46 (39%) were positive for HD, 6 (13%) were TP positive, 3 (7%) were positive for TP/HD, and 19 (41%) were negative for both pathogens tested. Healing rate was 80.4% (95% Confidence Interval [CI], 73.9–95.5) for all cases; 72.2% (95% CI: 57.0–93.4) for HD only, 83.3% (95% CI: 43.6–97.0) for TP only, 100% (95% CI: 43.9–100) for TP/HD, and 84.2% (95% CI: 62.4–94.5) for negative cases. Complete healing was generally observed by day 15–20.

**Conclusion:**

These results support the ongoing use of a single dose azithromycin in yaws elimination programmes, as evidenced by the healing rates observed. Nonetheless, greater focus should be directed toward improving diagnostic and treatment approaches for individual patients. Further research is necessary to better understand the aetiology of cutaneous ulcers in yaws-endemic regions.

## Author summary

Yaws is a tropical skin disease caused by the bacterium *Treponema pallidum* subsp. *pertenue*, which affects people in tropical regions like Ghana, and can lead to deformities without treatment. The disease is mostly found in children less than 15 years old and is transmitted by direct skin-to-skin contact with infectious lesions. Currently, it is treated with a single oral dose of 30mg/kg of Azithromycin. Diagnosis primarily relies on serological tests, with the WHO recommending the Dual Path Platform (DPP) Syphilis Screen and Confirm Assay. However, due to the high cost of DPP test kits, many countries depend on clinical diagnosis, which requires skilled health workers for accuracy. Given that *H. ducreyi* has been reported to cause lesions clinically resembling those of yaws, this overlap can hinder accurate diagnosis and may potentially influence the treatment outcomes for yaws. Our findings are important because they show how multiple organisms can complicate disease control, highlight the need for better diagnostic tools, and possibly adjust treatments to ensure everyone gets better. This could help health workers improve care and support the global effort to eliminate yaws, benefiting both local communities and global health.

## Introduction

Yaws, caused by *Treponema pallidum* subsp. *pertenue* (*T. pallidum* subsp *pertenue*) [1] is a chronic, neglected tropical disease (NTD) that primarily affects the skin, bones, and cartilage and is most prevalent among children under 15 years [2] in impoverished, rural communities in the tropics [3]. If not treated, the initial infectious papillomatous forms of the disease could form infectious ulcers that can quickly spread to affect other persons [4]. Transmitted through direct skin contact [4, 5], yaws is targeted for global eradication by 2030 through the World Health Organization’s (WHO) Morges Strategy, which relies heavily on mass drug administration (MDA) of single dose oral azithromycin (30mg/kg) and active case detection [6–8].

The aetiology of ulcerative skin lesions in yaws-endemic areas has become more complex in recent years. Studies using molecular tools have shown that *Haemophilus ducreyi (H. ducreyi)*, a bacterium historically associated with chancroid, has been implicated as a cause of about one-third of cutaneous skin ulcers among children living in yaws-endemic regions [9–15], and endemic communities in Ghana are not an exception [16–18]. These skin ulcers commonly affect children and occur on exposed areas such as the legs and arms. Transmission is thought to occur through direct skin contact or contaminated environments rather than sexual exposure [19].

The results of clinically yaws-like lesions, which are mono- or co-infected with *H. ducreyi,* have called into question the long-held belief that *T. pallidum* subsp. *pertenue* is the only cause of clinically suspected yaws cases in endemic areas. This highlights the need to understand the effects of *H. ducreyi* on yaws and yaws eradication efforts.

*Treponema pallidum (T. pallidum)* and *H. ducreyi (H. ducreyi)* skin ulcers are mostly not clinically distinguishable with certainty based on appearance alone [10, 11], and this has significant implications for disease surveillance, diagnosis, treatment outcomes, and eradication efforts, as *H. ducreyi* is known to cause cutaneous lesions in both sero-positive and sero-negative individuals within yaws-endemic communities [20].

Misclassification of cases could potentially lead to inflated yaws case counts (due to overdiagnosis) or an incorrect assumption of the number of azithromycin treatment failures due to yaws. Although both pathogens may respond to azithromycin [21–23], evidence suggests that *H. ducreyi* co-infections may resist standard azithromycin dosing and lead to persistent skin lesions [9, 24].

While the occurrence of *H. ducreyi* in yaws-like lesions have been widely documented [10, 11, 18], there is limited evidence regarding its influence on treatment outcomes assessed at the WHO-recommended 4-week follow-up. Understanding the influence of *H. ducreyi* on yaws cases can better inform diagnostic and treatment strategies to enhance complete lesion resolution, contributing to effective eradication efforts. This study aimed to detect the occurrence of *H. ducreyi* in yaws-confirmed cases in this study area using molecular diagnostics and assess its influence on yaws treatment outcomes.

## Methods

### Ethical statement

The study was approved by the Committee on Human Research, Publications and Ethics (CHRPE) of the School of Medical Sciences at the Kwame Nkrumah University of Science and Technology (CHRPE/AP/361/24 and CHRPE/AP/540/24). Permission was also sought from the school authorities and health management teams in the respective districts. Written informed consent was provided by parents or legal representatives of all participants. Additionally, participants aged 12–17 years provided written assent. All the study processes were conducted in accordance with the principles guiding research in human subjects as set out in the Declaration of Helsinki [25].

### Participants and study setting

From March to September 2024, we conducted a prospective cohort study of school children in communities from two yaws-endemic districts, Wassa Amenfi East and Aowin, in Ghana. Wassa Amenfi East and Aowin districts are neighboring administrative districts located in the southwestern part of Ghana, within the Western and Western North Regions respectively. Both districts are part of the forest zone of Ghana, and are characterized by high rainfall, dense vegetation, with primarily rural populations who are engaged mainly in farming and small-scale mining [26–28]. The Wassa Amenfi East district, with Wassa Akropong as the capital, has a population of 179,696, comprising 95,283 males and 84,413 females, with about 41% being children under 15 years [26]. The Aowin district, having Enchi as the capital, has a population of 129,721, made up of 68,236 males and 61,485 females, and approximately 40% of the population are children under 15 years [26].

### Participants’ recruitment and sampling

Potential participants were approached through a school-based yaws screening campaign in both districts with Community health workers (CHWs) and local health authorities facilitating initial engagement in villages, schools, and community centres. These sessions, using culturally sensitive communication and languages provided education about yaws, its symptoms, and the study’s objectives to encourage informed participation.

School-based screening was done in 33 selected schools in Wassa Amenfi East and 4 in Aowin districts. The selection of the schools was based on the recommendation of the respective District Health Management Teams (DHMT) using previous reporting patterns for yaws in the districts. All children present at the time of the school visit were clinically screened for yaws lesions: papilloma, ulcers, squamous macules, bone swellings, and palmar and plantar lesions as outlined in the WHO 2014 Yaws Pictorial Guide [29].

### Study procedures

The study team, comprising clinicians, researchers, and Disease Control Officers with experience in infectious diseases and public health, received supplemental training on the identification and management of yaws and yaws-like lesions as well as the study procedures prior to the field work. The training was provided by two Consultant Infectious Diseases Physicians (YAA and ROP) with long-standing experience in skin NTDs. A total body screening was done for all children present at the time of the school visit. Skin examinations were conducted using gender-appropriate procedures and focused on exposed body areas, including the feet, legs up to the knees, forearms/hands (with the students in school uniforms that consisted of above-knee shorts and short-sleeved shirts), neck, face, and scalp. Shoes and socks were removed before the skin examination. The examination excluded the breasts and genitals unless participants specifically requested their inclusion, in which case they were evaluated in a separate, private area. Individuals having yaws-like ulcers and a positive rapid point-of-care treponemal test (SD Bioline Syphilis 3.0 RDT kit, Standard Diagnostics Inc., Suwon, South Korea) and subsequently confirmed with a dual positive treponemal and nontreponemal test (DPP Syphilis Screen and Confirm Assay, Chembio Diagnostic Systems, Medford, NY, USA) [30] were identified. In this study, only participants with DPP positive yaws ulcers who had swabs from their ulcers collected for Polymerase Chain Reaction (PCR) were recruited.

Clinical features of the ulcers, such as the number, shape, size, tenderness, induration, colour, and location, were recorded. To determine ulcer shape and size, the largest and shortest diameters of the ulcers were measured in centimetres using a disposable ruler; circular/ regular shapes had a length-to-width ratio of approximately 1:1, and irregular shapes showed no consistent pattern. This was done to confirm the shape obtained through visual inspection. Tenderness was evaluated by applying gentle pressure on the ulcer margins and surrounding tissue with a gloved finger and classified as yes (present) or no (absent). The presence of pain was also assessed based on self-reporting by participants and an involuntary pain response during palpation. Induration was determined by palpating the ulcer margins with the fingertips and graded as yes (present) or no (absent). Colour was assessed under natural light, noting the base as uniform (entirely red granulation tissue) and non-uniform (mixed red and yellow areas), and recorded. An ulcer was defined as deep when it presented full thickness loss of the dermis and/or subcutaneous tissue (i.e., deep crater with or without undermining of adjacent tissue).

Lesions were photographed, and a dry, sterile lesional swab of each of the skin ulcers was collected. All participants with DPP positive yaws were treated with Azithromycin at a dose of 30mg/kg under direct observation of trained community nurses as recommended by the WHO [31] and followed up at 4 weeks post-treatment. In addition to antibiotic treatment, basic wound care was provided for participants, and participants were asked to document the day of complete healing. Participants were reviewed at week 4 post-antibiotic treatment by the survey team to assess the outcome of treatment. Photographs of ulcers were taken for comparison to assess clinical resolution by the two Consultant Physicians. Participants’ information was recorded on the Skin NTDs Clinical and Treatment form (SkinNTDs 01) (S1 File).

For analysis, lesion response to treatment was graded as completely healed or not completely healed based on clinical assessment by the two Consultant Physicians. A lesion was classified as completely healed if it exhibited full re-epithelialization with no residual exudate, crust, or open wound, and the surrounding skin appeared normal (e.g., no erythema or induration) under natural light. A lesion was classified as not completely healed if any open wound, exudate, crust, or persistent erythema/induration remained, or if new lesions appeared at the site and were recorded. This category included lesions that have had some improvement, no improvement, or deterioration (increase in size or emergence of new lesions) compared to that at baseline, as assessed on photography. Date of healing as reported by the participants were recorded, and lesion samples were taken from all unhealed ulcers at follow-up for PCR.

### Laboratory procedures

All laboratory assessments were carried out at the Kumasi Centre for Collaborative Research in Tropical Medicine (KCCR) in Ghana. Nucleic acid extraction and purification were done using the QIAamp DNA Mini Kit (Qiagen, Hilden, Germany). Briefly, samples underwent a process of Proteinase K lysis, purification on the QIAamp Mini spin columns, adsorption of genetic material to the QIAamp membrane, removal of residual contaminants by washing, and final elution of nucleic acid as per the manufacturer’s instructions [32]. The multiplex qPCR assay was performed using the RealCycler - universal kit (Progenie molecular, Valencia, Spain) for the detection of the PolA and HgbA specific genes for *T. pallidum* and *H. ducreyi,* respectively, on a Bio-Rad CFX96 Real-time PCR detection system (Bio-Rad Laboratories, Paris, France) according to manufacturer instructions and as previously described [18].

After PCR, participants were classified into four groups: (1) Participants with *T. pallidum* DNA only (TP only), (2) *H. ducreyi* DNA only (HD only), (3) *T. pallidum* and *H. ducreyi* DNA co-infection (TP/HD), and (4) participants who were negative for DNA of both *T. pallidum* and *H. ducreyi* (Negative).

### Statistical analysis

Data cleaning and necessary corrections were carried out manually during the collection process. Data was entered and managed with Microsoft Excel 2019 (Microsoft Corp., Redmond, Washington, USA). The cleaned data were checked for consistency and analyzed using RStudio version 2023.12.0.369 (RStudio: Integrated Development Environment for R. Posit Software, PBC, Boston, MA, USA) and SPSS statistics version 26 (IBM Corp., Armonk, NY, USA). All participant data were anonymized before analysis.

We estimated the prevalence of *H. ducreyi* (HD) as a cause of cutaneous ulcers in the study population using SPSS. Clinical characteristics of participants with TP only, HD only, TP/HD, and Negative ulcers were analyzed and compared using Fisher’s exact test for categorical variables and the Kruskal-Wallis test for continuous variables. Multinomial logistic regression was used for the odds ratio at 95% confidence interval (CI), comparing the clinical characteristics and outcomes across the aetiological groups using RStudio. A p value <0.05 was set as the level of statistical significance.

## Results

### Characteristics of the study population

While 71 participants with different clinical forms of yaws tested positive on the DPP test, results for the 46 participants with ulcers whose samples were collected for PCR are presented in this study. Three participants had ulcers, but samples could not be collected because they were too small, dry and crusted.

**Fig 1:**
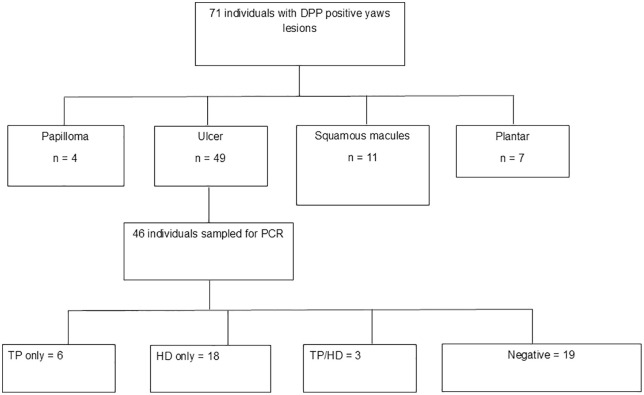
Flow Chart of study participants. **TP** only: Participants with ***T. pallidum* DNA** only; **HD** only: ***H. ducreyi* DNA** only; **TP/HD**: ***T. pallidum* and *H. ducreyi* DNA** co-infection; Negative: participants who were negative for **DNA** of both ***T. pallidum* and *H. ducreyi*.**

Of the 46 participants who underwent PCR testing, the prevalence of HD only was 18/46 (39%) after PCR. TP only was identified in 6 (13%) ulcers, TP/HD in 3 (7%), and 19 (41%) were negative for both pathogens tested (Negative) (Fig 1).

### Demographic and clinical characteristics

Table 1 summarizes the demographic and clinical characteristics of the study participants across the four diagnostic groups after PCR. The median age of all participants was 11 years (IQ: 8.00-12.75), and that of HD only was 10.50 (IQ: 9.25-12.00) with no significant differences (p = 0.087) between the groups. Males dominated all groups (78% overall, p > 0.9). There was a higher proportion of HD only ulcers from Aowin district (83%) than from Wassa Amenfi East (17%).

**Table 1:** Demographic and clinical characteristics of study participants.

Characteristic		TP only,	HD only,	TP/HD,	Negative,	Overall,	p-value^1^	TP vs HD,	TP/HD vs HD,	Negative vs HD,
N = 46		n (%)	n (%)	n (%)	n (%)	n (%)		OR (95% CI)	OR (95% CI)	OR (95% CI)
**Age, Median (IQR), years**		11.50 (8.75, 12.00)	10.50 (9.25, 12.00)	6.00 (6.00, 7.00)	11.00 (9.00, 13.50)	11.00 (8.00, 12.75)	0.09^α^	–	–	–
**Gender**	Male	5 (83)	14 (78)	2 (67)	15 (79)	36 (78)	>0.9	1.43 (0.13-16.03)	0.57 (0.04-8.05)	1.07 (0.22-5.13)
	Female	1 (17)	4 (22)	1 (33)	4 (21)	10 (22)		1	1	1
**Number of lesions**	Single	3 (50)	15 (83)	3 (100)	18 (95)	39 (85)	0.07	1	1	1
	Multiple	3 (50)	3 (17)	0 (0)	1 (5.3)	7 (15)		5.00 (0.66-37.85)	–	0.28 (0.03-2.96)
**Shape of lesion**	Round	4 (67)	12 (67)	3 (100)	10 (53)	29 (63)	**0.02**	1	1	1
	Irregular	2 (33)	6 (33)	0 (0)	9 (47)	17 (27)		0.70 (0.06-7.85)	–	3.15 (0.75-13.17)
**Smallest diameter**	<2 cm	4 (67)	16 (89)	2 (67)	15 (79)	37 (80)	0.50	0.25 (0.03-2.36)	0.25 (0.02-4.17)	0.47 (0.08-2.95)
	≥2 cm	2 (33)	2 (11)	1 (33)	4 (21)	9 (20)		1	1	1
**Largest diameter**	<2 cm	4 (67)	15 (83)	2 (67)	14 (74)	35 (76)	0.70	0.40 (0.05-3.27)	0.4 (0.03-5.96)	0.56 (0.11-2.79)
	≥2 cm	2 (33)	3 (17)	1 (33)	5 (26)	11 (24)		1	1	1
**Duration before healthcare**	<4weeks	5 (83)	7 (39)	2 (67)	8 (42)	22 (48)	>0.9	7.86 (0.75-82.13)	3.14 (0.24-41.51)	1.14 (0.31-4.25)
	≥4weeks	1 (17)	11 (61)	1 (33)	11 (58)	24 (52)		1	1	1
**Presence of pain**	Yes	1 (17)	13 (72)	2 (67)	7 (37)	23 (50)	**0.04**	0.08 (0.01-0.83)	0.77 (0.06-10.49)	0.22 (0.06-0.90)
	No	5 (83)	5 (28)	1 (33)	12 (63)	23 (50)		1	1	1
**Deep ulcer**	Yes	3 (50)	6 (33)	2 (67)	5 (26)	16 (35)	0.40	1	1	1
	No	3 (50)	12 (67)	1 (33)	14 (74)	30 (65)		5.00 (0.08-3.36)	0.25 (0.02-3.42)	1.40 (0.34-5.77)
**Indurated edges,**	Yes	2 (33)	12 (67)	2 (67)	4 (21)	20 (43)	**0.02**	1	1	1
	No	4 (67)	6 (33)	1 (33)	15 (79)	26 (57)		4.00 (0.56-28.40)	1.00 (0.08-13.37)	7.50 (1.72-32.80)
**Uniform colour,**	Yes	5 (83)	6 (33)	2 (67)	4 (21)	17 (37)	**0.02**	1	1	1
	No	1 (17)	12 (67)	1 (33)	15 (79)	29 (63)		0.10 (0.06, 0.01-1.06)	0.25 (0.02-3.34)	1.88 (0.43-8.20)
**Tenderness**	Yes	2 (33)	4 (22)	1 (33)	2 (11)	9 (20)	0.40	1.75 (0.59, 0.23-13.31)	1.75 (0.12-24.65)	0.41 (0.07-2.59)
	No	4 (67)	14 (78)	2 (67)	17 (89)	37 (80)		1	1	1
**District**	Aowin	3 (50)	15 (83)	2 (67)	13 (68)	33 (72)	0.40	0.20 (0.03-1.51)	0.40 (0.3-5.96)	0.43 (0.09-2.09)
	Wassa Amenfi East	3 (50)	3 (17)	1 (33)	6 (32)	13 (28)		1	1	1

HD, Haemophilus ducreyi; TP, Treponema pallidum; NEGATIVE, negative for TP and HD; CI, Confidence interval; OR, Odd Ratio; Data are presented as n (%) unless otherwise indicated; n, number of cases; IQR, interquartile range; p value^**1**^, Fisher’s exact test, ^α^Kruskal-Wallis rank sum test; OR, Odd Ratio calculated using multinomial logistic regression model.

HD only ulcers were predominantly single (83%), round (67%), less than 2 cm on the largest and smallest diameters (83 and 89%), and 11 (61%) participants reported having had the ulcers for more than 4 weeks. These ‘HD only’ ulcers were also found to be significantly indurated (67%) and superficial (67%). Compared to TP only ulcers, pain was significantly higher in the HD only group 13 (72%).

Among the participants, with TP only lesions, the ulcers were round (67%), mostly painless (83%), had uniform colour (83%), and deep (50%) and without indurated edges (67%).

### Assessment of treatment outcomes

**Fig 2:**
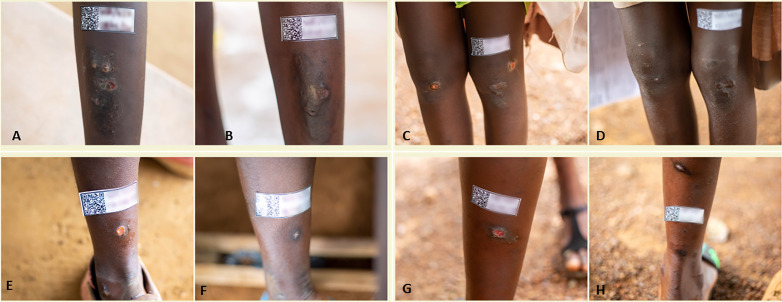
Assessment of treatment outcomes following a single-dose oral Azithromycin for ulcers caused by *T. pallidum* (A): before treatment and (B): post treatment; *H. ducreyi* (C): pretreatment and (D): post treatment; *T. pallidum* and *H. ducreyi* coinfection (E): pretreatment and (F): post treatment; Negative for *T. pallidum* and *H. ducreyi* (G): pretreatment and (H) post treatment. All post treatment assessments were undertaken 4 weeks after administration of single dose oral azithromycin.

Fig 2 shows the images of selected participants in different aetiologic groups before treatment and at week 4 post azithromycin therapy. There was a generally good outcomes following treatment with single dose oral azithromycin across all aetiologic groups. Thirty seven of the 46 (80.4%, 95% CI: 73.9–95.5%) participants had completely healed lesions.

The median age of participants with completely healed lesions was 11.00 years (IQR: 8.00–13.00), with no significant differences across aetiologic groups (p = 0.09). Age distribution showed 32% (n = 12) in the 5–9 years category and 68% (n = 25) in the 10–15 years category, with a trend toward younger age in TP/HD cases (100% aged 5–9, p = 0.10). Clinical features included round shape in 73% (n = 27, p = 0.30), pain in 49% (n = 18, p = 0.50), deep ulcers in 24% (n = 9, p = 0.20), indurated edges in 43% (n = 16, p = 0.03), and uniform colour in 35% (n = 13, p = 0.06), with indurated edges showing a significant association across groups. The characteristics of individuals with completely healed lesions are detailed in Table 2.

**Table 2:** Demographic and clinical characteristics of participants completely healed at follow-up.

Characteristic		TP only,	HD only,	TP/HD,	Negative,	Overall,	p-value^1^
n = 37		n = 5 (%)	n = 13 (%)	n = 3 (%)	n = 16 (%)	n = 37 (%)	
**Age, Median (IQR), years**		12.00	10.00	6.00	11.00	11.00	0.09^α^
		(11.00, 12.00)	(9.00, 12.00)	(6.00, 7.00)	(9.50, 13.25)	(8.00, 13.00)	
**Age category (years)**	5 – 9	1 (20)	4 (31)	3 (100)	4 (25)	12 (32)	0.10
	10 – 15	4 (80)	9 (69)	0 (0)	12 (75)	25 (68)	
**Gender**	Male	5 (100)	10 (77)	2 (67)	14 (88)	31 (84)	0.50
	Female	0 (0)	3 (23)	1 (33)	2 (13)	6 (16)	
**Number of lesions**	Single	3 (60)	11 (85)	3 (100)	16 (100)	33 (89)	0.07
	Multiple	2 (40)	2 (15)	0 (0)	0 (0)	4 (11)	
**Duration before healthcare**	<4weeks	4 (80)	6 (46)	2 (67)	7 (44)	19 (51)	0.60
	≥4weeks	1 (20)	7 (54)	1 (33)	9 (56)	18 (49)	
**Round shape**		4 (80)	11 (85)	3 (100)	9 (56)	27 (73)	0.30
**Presence of pain**		1 (20)	8 (62)	2 (67)	7 (44)	18 (49)	0.50
**Deep ulcer**		2 (40)	2 (15)	2 (67)	3 (19)	9 (24)	0.20
**Indurated edges**		2 (40)	9 (69)	2 (67)	3 (19)	16 (43)	0.03
**Uniform colour**		4 (80)	3 (23)	2 (67)	4 (25)	13 (35)	0.06
**District**	Aowin	3 (60)	10 (77)	2 (67)	10 (63)	25 (68)	0.80
	Wassa Amenfi East	2 (40)	3 (23)	1 (33)	6 (38)	12 (32)	

p-value^1^ = Fisher’s exact test: ^α^Kruskal-Wallis rank sum test.

The 19.6% (9/46; 95% CI, 4.5%-28.5%), in the not completely healed group at follow-up (Table 4), comprised participants with some improvement, no improvement, or deteriorated (increase in lesion size or new lesions) ulcers. This included 27.8% (5/18; 95% CI: 6.6–43.0%) as HD only, 16.7% (1/6; 95% CI: 3.0–56.4%) as TP only, 0% (95% CI: 0–56.1%) as TP/HD, and 15.8% (3/19; 95% CI: 5.5–37.6%) were negative (Tables 3 and 4). HD-only recorded the highest number of cases (5/9; 56%) among the ‘not completely healed’ group. Pain was reported in 56% of cases (5/9), all being HD only ulcers and was statistically significant (p = 0.01). Eight of the nine participants (88.9%) had partially healed ulcers, and 1 (11.1%) had no improvement at follow-up. Swab samples taken from all ulcers classified as not completely healed at follow-up for molecular analysis were negative for *T. pallidum* and *H. ducreyi*. Out of the participants not completely healed, the highest number was from the HD only group (5/9; 56%), with all having painful ulcers.

**Table 3:** Demographic and clinical characteristics of participants not completely healed at follow-up.

Characteristic		TP only,	HD only,	TP/HD,	Negative,	Overall,	p-value
N = 9		N = 1 (%)	N = 5 (%)	N = 0	N = 3 (%)	N = 9 (%)	
**Age, Median (IQR), years**						11.00	
						(8.50, 12.00)	
**Age category (years)**	5 – 9	1 (100)	1 (20)	–	1 (33)	3 (33)	0.60
	10 – 15	0 (0)	4 (80)	–	2 (67)	6 (67)	
**Gender**	Male	0 (0)	4 (80)	–	1 (33)	5 (56)	0.30
	Female	1 (100)	1 (20)	–	2 (67)	4 (44)	
**Number of lesions**	Single	0 (0)	4 (80)	–	2 (67)	6 (67)	0.60
	Multiple	1 (100)	1 (20)	–	1 (33)	3 (33)	
**Duration before healthcare**	<4weeks	1 (100)	1 (20)	–	1 (33)	3 (33)	0.60
	≥4weeks	0 (0)	4 (80)	–	2 (67)	6 (67)	
**Round shape**		1(100)	3 (60)	–	1 (33)	5 (56)	>0.90
**Presence of pain**		0 (0)	5 (100)	–	0 (0)	5 (56)	0.01
**Deep ulcer**		1 (100)	4 (80)	–	2 (67)	7 (78)	>0.90
**Indurated edges**		0 (0)	3 (60)	–	1 (33)	4 (44)	>0.90
**Uniform colour**		1(100)	3 (60)	–	0 (0)	4 (44)	0.20
**District**	Aowin	0 (0)	5 (100)	–	3 (100)	8 (89)	0.11
	Wassa Amenfi East	1 (100)	0 (0)	–	0 (0)	1 (11)	

p value = Fisher’s exact test.

**Table 4:** Clinical healing rates of *H. ducreyi*, *T. pallidum*, and PCR negative, DPP positive yaws ulcers at 4 weeks post-treatment.

Outcome	HD only	TP only	TP/HD	Negative	Overall	HD only vs TP only,	HD only vs TP/HD,	HD only vs Negative,
	(n = 18), % (95% CI)	(n = 6), % (95% CI)	(n = 3), % (95% CI)	(n = 19), % (95% CI)	(n = 46), % (95% CI)	OR (95% CI)	OR (95% CI)	OR (95% CI)
**Completely Healed**	(13), 72.2 (57.0 - 93.4)	(5), 83.3 (43.6 - 97.0)	(3) 100 (43.9 - 100)	(16), 84.2 (62.4 - 94.5)	(37) 80.4 (73.9 - 95.5)	1.92 (0.18 - 20.8)	3.67 (NA)	2.05 (0.41 - 10.2)
**Not completely healed**	(5), 27.8 (6.6 - 43.0)	(1), 16.7 (3.0 - 56.4)	(0), 0 (0 - 56.1)	(3), 15.8 (5.5 - 37.6)	(9), 19.6 (4.5 - 28.5)	0.52 (0.05-5.6)	0.0 (NA)	0.49 (0.1-2.4)

Among cases with HD only ulcers, 72.2% (13/18; 95% CI: 57.0–93.4%) achieved complete healing, compared to 83.3% (5/6; 95% CI: 43.6–97.0) for TP only, 100% (95% CI: 43.9–100) for TP/HD, and 84.2% (16/19; 95% CI: 62.4–94.5) for negative cases. The odds for complete healing in HD-only cases versus TP-only cases was 1.92 (95% CI: 0.2–20.8, p = 0.6), versus TP/HD was 3.67 (95% CI: NA, p > 0.9), and versus negative cases was 2.05 (95% CI: 0.4–10.2, p = 0.4), indicating no statistically significant differences between groups. The OR for non-healing in HD-only cases versus TP-only was 0.52 (95% CI: 0.1–5.6, p = 0.6), versus TP/HD was 0.0 (95% CI: NA, p > 0.9), and versus negative cases was 0.49 (95% CI: 0.1–2.4, p = 0.4), also showing no significant differences (Table 4).

At week 4 follow, samples from all the non-healed lesions were PCR negative for HD and TP.

**Fig 3:**
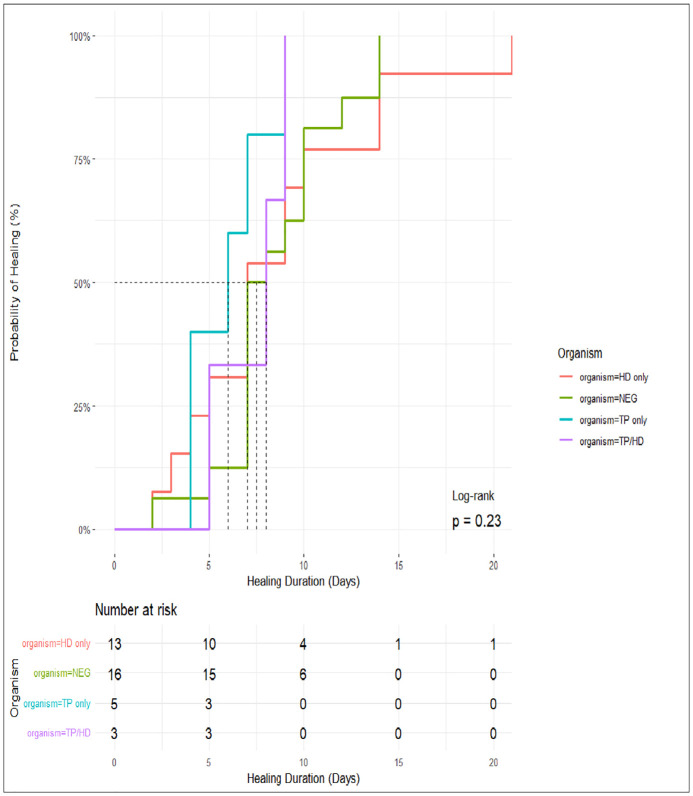
Kaplan-Meier estimate of healing probability over time by organism detection status.

A log-rank test comparing survival distributions revealed no statistically significant difference between groups (p = 0.23) indicating that healing trajectories were broadly similar across groups and time to healing did not significantly vary by detected organism (Fig 3). Some participants reported complete healing as early as day 2 post treatment, with a marked reduction in the number at risk by day 10. The time to reach 50% probability of healing occurred between 6 and 8 days across the different organism categories, with complete healing generally observed by day 15–20. By day 9, all the participants in the TP only and TP/HD groups had healed completely, with one participant in the HD only group still at risk by day 20.

## Discussion

This study found *H. ducreyi* as a common organism in cutaneous ulcers among individuals in a yaws-endemic region, Ghana. This finding is consistent with findings worldwide that have reported a substantial proportion of DPP positive yaws ulcers testing positive for *H. ducreyi* via PCR, [9, 18]. In our cohort, *H. ducreyi* was identified as the sole pathogen in 18 (39.1%) of DPP-positive yaws ulcers and as a co-infection with *T. pallidum* in 3 (6.5%) of individuals, resulting in a combined prevalence of 45.6% for HD only and TP/HD co-infections. This high proportion underscores *H. ducreyi*’s significant role as a major contributor to cutaneous ulcers in yaws-endemic areas, complicating clinical and serological diagnosis due to overlapping clinical presentations with yaws, as observed in endemic regions.

Our study enhances the understanding of the clinical characteristics of cutaneous ulcers in yaws-endemic regions by describing features of *H. ducreyi* (HD) only and *T. pallidum* (TP) only ulcers. HD-only ulcers were typically superficial (67%), small in both largest (83%) and shortest (89%) diameters, painful (72%) and lacked induration (61%). These findings align with observations from other yaws-endemic tropical regions, such as Papua New Guinea, where HD-associated cutaneous ulcers are often described as shallow and small, reflecting a less invasive inflammatory process compared to treponemal infections [21]. In contrast, TP-only ulcers were predominantly round (67%), small (<2 cm in 75%, for both diameters), painless (83%), tender (67%), and uniform in color (67%), underscoring their inflammatory nature. These characteristics partially align with studies, where TP only yaws are noted for their round shape and less painful ulcers [21, 24]. The predominance of smaller lesions (<2 cm) across all groups suggests that ulcer size, as measured by the largest and smallest in this area, is not a distinguishing feature among the aetiological agents. Although there was no statistical significance in size across the groups, on physical examination, most of the ‘HD only’ ulcers were smaller than the TP only ulcers.

The findings on healing rates for our study contribute to the growing evidence that *H. ducreyi* (HD) in cutaneous ulcers is effectively treated with azithromycin [21, 33], as all individuals with unhealed lesions at follow-up samples tested PCR negative for HD. Persistent cutaneous HD ulcers may not be due to azithromycin drug response or resistance. However, incomplete healing in HD-only ulcers may be attributed to several factors not investigated in this study, including secondary bacterial colonization, slow host-dependent tissue healing, or non-infectious causes such as trauma, given that children in these yaws-endemic areas frequently engage in contact sports, farming, and other activities that may cause skin abrasions and impair healing, particularly since they often leave wounds uncovered. As reported by other studies, the ubiquity of *H. ducreyi* may contribute to the incomplete healing in the HD only ulcers in the study area [34]. Although not statistically significant (p > 0.9), males dominated across all groups (78% overall, 83% in TP only) as elsewhere [35], likely due to their greater involvement in physical contact games, farming tasks, and other activities in these rural endemic areas, which may lead to minor skin abrasions that facilitate the transmission of yaws and *H. ducreyi* infection.

The uniform healing by day 15 (97.3%) across all groups supports the WHO’s Morges strategy of using azithromycin MDA for yaws eradication, as it appears effective across all the aetiological agents in this setting. The TP only and TP/HD groups healed faster by day 9, though no significant difference in healing times was observed (log-rank p = 0.23). The rapid healing in HD cases aligns with azithromycin’s efficacy against *H. ducreyi* [21]*,* although complete healing was achieved at day 21.

This study highlights the complexity of diagnosing cutaneous ulcers in yaws-endemic settings, where *T. pallidum* subsp. *pertenue*, *H. ducreyi*, and coinfections present with overlapping but distinct clinical features. Lesion shape, pain, and color emerged as potential diagnostic markers, but molecular confirmation remains critical to avoid misclassification. These findings support the continued use of a single dose of azithromycin in yaws eradication efforts while emphasizing the need for enhanced diagnostic tools and further and larger studies to clarify the epidemiology of cutaneous ulcers in yaws-endemic regions. coinfections. To achieve eradication, there is the need for continuous surveillance, and improved wound care practices such as use of wound dressings or coverings to minimize contamination and traumatization that may delay healing or complicate the lesions. Molecular tools such as PCR will be useful for post elimination surveillance and monitoring of azithromycin resistance.

The Dual Path Platform (DPP) rapid serologic test for yaws, which detects both treponemal and non-treponemal antibodies, shows good performance compared with standard serology but more limited agreement with *Treponema pallidum* subsp. *pertenue* PCR, the molecular reference for active infection. Meta-analyses report pooled sensitivity of the DPP treponemal component around 86% and non-treponemal 80%, with high specificity (96–97%) versus serological reference tests for yaws [36, 37]. In a study of clinically suspected ulcers, DPP detected up to 84% of PCR-confirmed yaws cases using the non-treponemal line read by eye, but specificity was modest, and combined test cut-offs showed 75–78% sensitivity and specificity against PCR [38]. These differences reflect the fact that serologic assays measure host antibody responses, while PCR detects pathogen DNA, so DPP may miss early or latent infections and yield false positives from past exposure. Furthermore, different disease phases such as incubation period or latency, or other confounders such as syphilis infections, may contribute to conflicting PCR and DPP results [38] as observed in the current study.

In 41.3% of individuals, no causative agent was identified on PCR, despite a significant number of these ulcers showing healing at follow-up. This situation warrants close monitoring, as it could pose a potential threat to eradication efforts. These ulcers might be non-infectious, possibly resulting from trauma or insect bites, or caused by organisms not detected by the current PCR assay, and could undermine eradication goals in a manner similar to *H. ducreyi* infection.

The study had some limitations. First was the relatively small sample size which led to the low statistical power in the analysis. We did not assess long-term follow-up beyond the 4 weeks to assess healing and healing dates for the not completely healed participants. In this study, we assessed healing time at the recommended week 4 post antibiotic administration follow visit. We did not factor in the period prior to health seeking the estimation of time to healing. In addition, the conduct of the study among school children meant there were no participants ≥15 years, potentially missing adults with yaws. Future studies should use larger cohorts across all age groups and conduct follow-up at 6 and 12 months to assess complete healing times, recurrence, and possible azithromycin resistance using molecular tools in yaws endemic areas.

## Conclusion

*H ducreyi* is an important cause of cutaneous ulcers in yaws endemic regions. The use of single dose azithromycin led to good healing rates among cases, a finding that supports the ongoing use of a single dose azithromycin in yaws elimination programmes. Nonetheless, greater focus should be directed toward improving diagnostic and treatment approaches for individual patients. Although yaws is predominantly found in the < 15 years age group, to achieve eradication, all age groups should be included in future studies to avoid potential latent cases and resurgence of the disease. Further research is needed to better understand the aetiology of cutaneous ulcers in yaws-endemic regions.

## Supporting information

S1 File Skin NTDs Clinical and treatment form (SKINNTDS 01). (PDF)
