# Supplementary material for: Assessment of the role of Haemophilus ducreyi coinfection on outcomes of Yaws treatment in the southwestern part of Ghana
Source: PLoS Negl Trop Dis. 2026 Mar 30;20(3):e0014182. doi: 10.1371/journal.pntd.0014182 (PMC13046261; doi:10.1371/journal.pntd.0014182)
Supplement: S1 File — (PDF) [file pntd.0014182.s001.pdf]

Skin NTDs clinical and treatment form

Health facility : ____ Name of health worker treating patient: ________________________

Name of patient: ______________________	ID#: ______________	Date of birth (dd/mm/yyyy) : _ _ / _ _ / _ _ _ _ Age (years): _______
Country: ______________________	District: ______________	Sex: ☐ Male ☐ Female
Province/Region/State: ______________________	Village/town: ______________	Phone number: ____________ Occupation: ______________________
Landmark: ______________________	GPS Coordinates:	Contact name: ______________ Contact phone: ______________

**HISTORY AT ADMISSION**	
**Mode of detection:**	☐ Active screening ☐ Contact tracing ☐ Passive/voluntary ☐ School survey ☐ Transfer in ☐ Other: ________
**Classification of patient:**	☐ New ☐ Recurrent/relapse ☐ Retreatment

Duration of illness before seeking care (weeks): _________		Previous treatment of current lesion(s)	☐ Yes *Specify*: ______________ ☐ No<br>Duration (days): _____________
Use of traditional treatment	☐ Yes ☐ No		
Family member or close contact with similar lesion(s)	☐ Yes ☐ No<br>Relationship: ______________<br>Diagnosis :______________	Treatment for previous lesion(s) *(if recurrent)*	☐ Yes *Specify*: ______________ ☐ No<br>Duration (days): _____________

REFERRED BY:

☐ Self-referral	☐ Former patient
☐ Health worker (HW)	☐ Schoolteacher
☐ Village HW	☐ Other (specify)
☐ Family member	_____________

INITIAL CLINICAL EXAMINATION

Date of clinical examination (dd/mm/yyyy) : _ _ / _ _ /_ _ _ _

BCG: ☐ Scar seen ☐ Scar dubious ☐ No scar

Weight (kg): [_____]

Pregnant: ☐ Yes ☐ No ☐ Unknown

HIV status: ☐ Positive ☐ Negative ☐ Unknown

**LIMITATION**	Limitation of movement (at any joint) ☐ Yes ☐ No	Limitation of activities: ☐ Yes ☐ No

**TYPE OF LESION(S)/ SWELLING**	☐ Macule (M)	☐ Oedema (E)	☐ Papilloma (Pa)	☐ Plaque (Q)	☐ Ulcer (U)	☐ Deformity (D)
	☐ Nodule (N)	☐ Osteomyelitis (O)	☐ Papule (P)	☐ Skin patches(S)	☐ Vesicles (V)	_____________

**LOCATION OF LESION(S)/ SWELLING**	☐ Abdomen (AB)	☐ Head and neck (HN)	☐ Lower limb (LL)	☐ Buttocks and perineum (BP)
	☐ Back (BK)	☐ ***Ear*****	☐ ***Toe*****	☐ ***Genitalia****
	☐ ***Breast (BR)****	☐ ***Eye****	☐ Upper limb (UL)	☐ Inguinal/Groin
	☐ Thorax (TH)	☐ ***Face*****	☐ ***Finger*****	

No. of lesions: [______]

Diameter of biggest lesion: [___] cm / [___] cm

**Pruritus:**	☐ Yes	☐ No
**Sensory loss:**	☐ Yes	☐ No
**Nerve tenderness:**	☐ Yes	☐ No
**Nerve enlargement:**	☐ Yes	☐ No
**Motor function loss:**	☐ Yes	☐ No

CLINICAL SUSPICION

☐ **BU**	☐ **Category I**: Single lesion, ≤ 5 cm in diameter	☐ **Category II** : Single lesion, 5–15 cm in diameter	☐ **Category III** : Single lesion > 15 cm in diameter, multiple lesions, lesions at critical sites, osteomyelitis
☐ **CL**	☐ **Situation I:** < 4 lesion(s), < 4 cm in diameter, not potentially disfiguring, infected with *Leishmania major*, <u>not</u> immunocompromised	☐ **Situation II**: < 4 lesions, < 4 cm in diameter, locally treatable, infected with *L. infantum* or *L. tropica*, <u>not</u> immunocompromised	☐ **Situation III**: ≥ 4 lesions, ≥ 4 cm in diameter, not compatible with local treatment, immunocompromised or suffering from unbalanced diabetes
☐ **Leprosy**	☐ **Paucibacillary:** ≤ 5 lesions	☐ **Multibacillary:** > 5 lesions	
☐ **Lymphatic filariasis**			
☐ **Yaws**			
☐ **Other**	Please specify: ____		

**** Critical site for Buruli ulcer*** ***** Critical site for cutaneous leishmaniasis***

LABORATORY CONFIRMATION

**Specimen(s) collected:** ☐ **Yes** ☐ **No**<br>**Date first specimen(s) taken** dd / mm / yyyy;	**Specimen type(s):**<br>☐ Biopsy ☐ Blood ☐ Fine needle aspiration (FNA) ☐ Slit skin smear ☐ Swab ☐ Urine

	Type of test	*Date initial test*	*Initial result*	*Date repeated test*	*Repeated result*
☐ **BU**	☐ **Ziehl–Neelsen:**	dd-mm-yyyy	☐ *Positive* ☐ *Negative* ☐ *Inconclusive*	dd-mm-yyyy	☐ *Positive* ☐ *Negative* ☐ *Inconclusive*
	☐ **Histology:**	dd-mm-yyyy	☐ *Positive* ☐ *Negative* ☐ *Inconclusive*	dd-mm-yyyy	☐ *Positive* ☐ *Negative* ☐ *Inconclusive*
	☐ **Mycolactone:**	dd-mm-yyyy	☐ *Positive* ☐ *Negative* ☐ *Inconclusive*	dd-mm-yyyy	☐ *Positive* ☐ *Negative* ☐ *Inconclusive*
	☐ **PCR:**	dd-mm-yyyy	☐ *Positive* ☐ *Negative* ☐ *Inconclusive*	dd-mm-yyyy	☐ *Positive* ☐ *Negative* ☐ *Inconclusive*
	☐ **Culture:**	dd-mm-yyyy	☐ *Positive* ☐ *Negative* ☐ *Inconclusive*	dd-mm-yyyy	☐ *Positive* ☐ *Negative* ☐ *Inconclusive*

	Type of test	*Date initial test*	*Initial result*	*Date repeated test*	*Repeated result*
☐ **CL**	☐ **Culture:**	dd-mm-yyyy	☐ *Positive* ☐ *Negative* ☐ *Inconclusive*	dd-mm-yyyy	☐ *Positive* ☐ *Negative* ☐ *Inconclusive*
	☐ **Direct exam:**	dd-mm-yyyy	☐ *Positive* ☐ *Negative* ☐ *Inconclusive*	dd-mm-yyyy	☐ *Positive* ☐ *Negative* ☐ *Inconclusive*

	Type of test	*Date initial test*	*Initial result*	*Date repeated test*	*Repeated result*
☐ **Leprosy**	☐ **Skin smear:**	dd-mm-yyyy	☐ *Positive* ☐ *Negative* ☐ *Inconclusive*	dd-mm-yyyy	☐ *Positive* ☐ *Negative* ☐ *Inconclusive*
	☐ **AMR* test:**	dd-mm-yyyy	☐ *Susceptible* ☐ *Resistant*	*If resistant, specify:* ☐ *Rifampicin* ☐ *Dapsone*	☐ *Rifampicin and Dapsone* ☐ *Ofloxacin*

	Type of test	*Date initial test*	*Visible lines*	*Date repeated test*	*Visible lines*
☐ **Yaws**	☐ **RDT:**	dd-mm-yyyy	☐ *Control* ☐ *Trep line*	dd-mm-yyyy	☐ *Control* ☐ *Trep line*
	☐ **DPP:**	dd-mm-yyyy	☐ *Control* ☐ *Trep line* ☐ *Non Trep line*	dd-mm-yyyy	☐ *Control* ☐ *Trep line* ☐ *Non Trep line*
	☐ **PCR:**	dd-mm-yyyy	☐ *Positive* ☐ *Negative* ☐ *Inconclusive*	dd-mm-yyyy	☐ *Positive* ☐ *Negative* ☐ *Inconclusive*

OTHER LABORATORY TESTS

Type of test	*Date initial test*	*Initial result*	*Date repeated test*	*Repeated result*
☐ **HIV test**	dd-mm-yyyy	☐ *Positive* ☐ *Negative* ☐ *Inconclusive*	dd-mm-yyyy	☐ *Positive* ☐ *Negative* ☐ *Inconclusive*
☐ **Pregnancy test**	dd-mm-yyyy	☐ *Positive* ☐ *Negative* ☐ *Inconclusive* ☐ *Not applicable*		
☐ **Other test**	dd-mm-yyyy	*Please specify tests and results:*		

FINAL CLINICAL DIAGNOSIS

☐ **Buruli ulcer** ☐ **Cutaneous leishmaniasis** *Parasite :* ________________ ☐ **Leprosy MB** ☐ **Leprosy PB** ☐ **Lymphatic filariasis** ☐ **Yaws** ☐ **Other (*specify*):** ____________________ ____________________________________

*** AMR, antimicrobial resistance**

FOR BURULI ULCER CASES

**TREATMENT**	Date treatment started: _ _ /_ _ / _ _ _ _ Was the patient hospitalized? ☐ Yes ☐ No Date of admission (if applicable): _ _ /_ _ / _ _ _ _
**TREATMENT PLAN** *(Tick all applicable)*	☐ Wound management ☐ Antibiotics ☐ POD (prevention of disability) ☐ Surgery (date: __ __/__ __/_ _ _ _) ☐ Rehabilitation
**ANTIBIOTIC (AB) TREATMENT**	☐ **Rifampicin: ________ (mg)** ☐ **Clarithromycin: ________ (mg)** ☐ **Streptomycin :_____ (mg)** ☐ **Other (name) : ______________ : ______ (mg)**

DOSAGE GUIDE

Weight of patient (kg)	Rifampicin			Clarithromycin		Streptomycin (1 g/2 ml)	
	Dose (mg)	Number of tablets (300 mg/tablet)	Number of tablets (150 mg/tablet)	Dose (g)	Number of tablets (500 mg/tablet)	Dose (g)	Volume (mL)
5–10	75	0.25	0.5			0.25	0.50
11–20	150	0.50	1.0			0.33	0.70
21–39	300	1.00	2.0			0.50	1.00
40–54	450	1.50	3.0			0.75	1.50
> 54	600	2.00	4.0			1.00	2.00

If streptomycin is contraindicated (e.g. pregnancy, previous treatment with streptomycin), please contact the national programme manager or a designated referral treatment centre.

DIRECTLY OBSERVED TREATMENT (DOT) *Cross out each day (X) after administering the antibiotics; if antibiotics are not taken, indicate with the symbol Ø*

Day / Month	1	2	3	4	5	6	7	8	9	10	11	12	13	14	15	16	17	18	19	20	21	22	23	24	25	26	27	28	29	30	31	Total Doses

**END OF AB TREATMENT**	**Date of AB treatment assessment** (dd/mm/yyyy) : _ _ / _ _ /_ _ _ _	**Date of AB treatment assessment** (dd/mm/yyyy) : _ _ / _ _ /_ _ _ _
**Serious adverse event**	☐ Yes *Specify*: ____	☐ No
**Antibiotics completed:**	☐ Yes ☐ No, defaulter ☐ No, medical reason	*If no,* number of days the antibiotics was taken: [____]<br>*If yes,* number of days missed ? [____] Longest gap (days) [____]

**TREATMENT OUTCOME**	**Date of treatment outcome assessment** (dd/mm/yyyy) : _ _ / _ _ /_ _ _ _
**Treatment outcome**	☐ Healed ☐ Referred ☐ Lost to follow-up ☐ Died
If healed*, *please specify	Date of discharge (dd/mm/yyyy): _ _ / _ _ / _ _ _ _ Date of complete healing (dd/mm/yyyy): _ _ / _ _ / _ _ _ _<br>Healed with surgery: ☐ Yes ☐ No Healed with joint limitation: ☐ Yes ☐ No Healed with scar: ☐ Yes ☐ No

FOR CUTANEOUS LEISHMANIASIS

**TREATMENT ROUTE** (tick all applicable):	☐ Wound management<br>☐ Cryotherapy	☐ Antiparasitic intralesional<br>☐ Thermotherapy	☐ Antiparasitic intramuscular/systemic<br>☐ Other: ______________________	
**TREATMENT MEDICINE**	**Sodium stibogluconate: ________ (mg)**	**Glucantime:_________ (g)**	**Other (name): ________________ : _________ (mg)**	
	**Number of doses: _____**	**Treatment start date: _ _ / _ _ /_ _ _ _**	**Treatment end date: _ _ / _ _ / _ _ _ _**	

**TREATMENT OUTCOME**							
**INITIAL**	**Date (dd/mm/yyyy): _ _ /_ _ /_ _ _ _**	**Severe adverse event:**	☐ Yes *Specify*: ____			☐ No	
		**Treatment completed:**	☐ Yes	☐ No, defaulter	☐ No, medical reason		
		**Initial treatment outcome:**	☐ Initial cure	☐ Failure	☐ Death	☐ Lost to follow-up	
**FINAL (D45-D90)**	**Date (dd/mm/yyyy): _ _ /_ _ /_ _ _ _**	**Final treatment outcome:**	☐ Cure	☐ Relapse	☐ Death	☐ Lost to follow-up	

FOR YAWS

**TREATMENT**	**Azithromycin :** ☐ Yes ☐ No	**Treatment date** (dd/mm/yyyy)**:** _ _ / _ _ /_ _ _ _
	**Dose:**	**Number of tablets:** _____
	**Serious adverse event:** ☐ Yes *Specify*:____ ☐ No	

**TREATMENT OUTCOME**<br><br>**(4 weeks)**	**Date (dd/mm/yyyy):** _ _ /_ _ /_ _ _ _
	☐ Completely healed ☐ Partially healed ☐ No improvement ☐ New lesion(s) ☐ Lost to follow-up

**DOSAGE GUIDE**			
**Age of patient (years)**	**Azithromycin**		
	**Total dose (mg)**	**Number of tablets**	**Syrup (ml)**
6 mths – 5 yrs	500	1	12.5
6–9	1000	2	
10–14	1500	3	
≥ 15	2000	4	

FOLLOW-UP APPOINTMENTS AFTER TREATMENT	
DATE (dd/mm/yyyy)	COMMENTS
___/___/______	
___/___/______	
___/___/______	
___/___/______	
___/___/______	
___/___/______	
___/___/______	
___/___/______	
